# Increasing skin-to-skin care in very and extremely preterm infants using an iterative quality improvement approach

**DOI:** 10.1016/j.jnn.2025.101681

**Published:** 2025-05-06

**Authors:** Tiana T. Nguyen, Matthew J. Miller, Elizabeth E. Rogers, Laurel Pershall, Jeannie Chan, Taylor Park, Diana Rogosa, Katelin Kramer

**Affiliations:** a Department of Physical Therapy and Rehabilitation Sciences, University of California, San Francisco, CA, USA; b Department of Pediatrics, Division of Neonatology, University of California, San Francisco, CA, USA; c UCSF Benioff Children’s Hospital, San Francisco, CA, USA

**Keywords:** Kangaroo care, Developmental rounds, Caregiver education, SENSE program

## Abstract

**Objective:**

This quality improvement (QI) initiative aimed to increase skin-to-skin care (SSC) and positive parental touch (PPT) rates to >90 % among infants <32 weeks’ gestation in a Level IV NICU.

**Methods:**

Guided by a key driver diagram, interventions were implemented through three Plan-Do-Study-Act (PDSA) cycles: developmental rounds (month 1), parent education (month 6), and the Supporting and Enhancing NICU Sensory Experiences program (month 8). We evaluated PPT within 72 h of birth, SSC within the first week of life, and time to first SSC during the development, implementation, and sustainability phases.

**Results:**

SSC rates increased from 71 % (development) to 93 % (implementation) and 94 % (sustainability). PPT rates sustained at 98 % during the implementation and sustainability phases. No increases in unplanned extubation or intraventricular hemorrhage were observed.

**Conclusion:**

A structured, multidisciplinary approach improved and sustained SSC rates for preterm infants without adverse effects.

## 1. Introduction

One in 10 infants in the United States is born preterm (less than 37 weeks) (Centers for Disease Control and Prevention, 2024). Preterm infants born at earlier gestational ages and with lower birth weights are at a significantly higher risk for serious developmental delays and disabilities (Centers for Disease Control and Prevention, 2024). While technological and medical advancements have greatly improved survival rates for preterm infants (Stoll et al., 2015), there remains a critical need to address the neurodevelopmental sequelae that can impact an infant and family’s long-term health and functioning (Pineda et al., 2013).

Preterm infants are often separated from their parents at birth due to the necessity of medical interventions in the neonatal intensive care unit (NICU). This separation disrupts the parent-infant bonding process, which is fundamental to an infant’s development. Positive parental touch (PPT), which includes providing containment with hand hugs, holding, and holding for skin-to-skin care (SSC), promotes parent-infant bonding and improves infant and maternal health outcomes, such as infant neurodevelopment, infant feeding, infant physiological stability, and maternal well-being (Clarke-Sather et al., 2024; Cong et al., 2021; Kucukoglu et al., 2021; Pados and Hess, 2020; Pineda et al., 2018). PPT can be initiated even earlier than SSC, as caregivers can provide hand hugs without the need to transfer infants out of the isolette.

Despite the well-documented benefits of SSC and PPT, infants born at lower gestational ages or with greater medical complexities often experience delayed and inconsistent SSC. Barriers to SSC implementation include healthcare provider concerns (e.g., risk of unplanned extubation [UPE] or intraventricular hemorrhage [IVH]), institutional challenges (e.g., limited staff training), and infant-specific medical instability (Linnér et al., 2022; Suitor, 2023). The gap identified in the literature highlights the need for targeted quality improvement (QI) initiatives to improve SSC practices for very and extremely preterm infants. As PPT can be initiated earlier and with fewer resources than SSC, it makes it an important immediate and complementary intervention.

Recognizing the crucial role of SSC and PPT in protecting and promoting the development of the preterm neonatal brain, we developed a QI initiative focused on increasing SSC and PPT for infants <32 weeks gestation at the University of California, San Francisco (UCSF) Benioff Children’s Hospital (BCH) NICU in conjunction and with the supportive structure of the California Perinatal Quality Care Collaborative (CPQCC) NICUs Enabling Optimal Brain Health (NEOBrain) QI collaborative (California Perinatal Quality Care Collaborative, nd). In addition to developing and implementing our QI initiative, we evaluated its effectiveness and assessed sustainability one-year post-implementation among infants <32 weeks gestation.

## 2. Methods

### 2.1. Quality improvement site

The UCSF BCH is a children’s hospital located in San Francisco, California with a certified regional 58-bed, Level IV NICU with an associated birthing hospital that serves infants throughout Northern California. The UCSF BCH uses a multi-disciplinary approach to provide advanced care to critically ill infants who were born preterm or with cardiopulmonary anomalies, gastrointestinal disorders, neurological disorders, and other illnesses requiring advanced life support. The UCSF BCH has over 900 admissions a year to the NICU and approximately 35 % of those admissions are due to preterm birth. There is a dedicated small baby unit with a specially trained team of neonatologists, nurse practitioners, nurses, physical and occupational therapists, respiratory therapists, and dieticians caring for infants <28 weeks gestation.

In April 2022, UCSF BCH NICU joined NEOBrain, a multi-center quality improvement (QI) collaborative hosted by the CPQCC, aimed at improving neuroprotective care for very and extremely preterm infants. We created a multidisciplinary QI team that consists of neonatologists, a neonatal nurse practitioner, bedside neonatal nurses, a clinical nurse specialist, neonatal occupational and physical therapists, a child life specialist, and a NICU parent liaison to guide the development and implementation of our SSC and PPT QI initiative.

### 2.2. QI initiative development, implementation, and sustainability measures

Our QI initiative targeted preterm infants born <32 weeks gestation because they are particularly susceptible to adverse neurodevelopmental outcomes (Saigal and Doyle, 2008). This QI initiative included infants admitted to the UCSF BCH NICU between December 2022 and June 2024 and was executed in three phases: development (December 2021 to May 2022), implementation (June 2022 to June 2023), and sustainability (July 2023 to May 2024). Infants were excluded from the QI initiative if they did not have a caregiver identified, expired during the first week of life, were transferred to UCSF after 7 days old, or had a caregiver unable to visit due to illness (e.g., COVID-19) or an intensive care unit admission. We also excluded infants who required high-frequency oscillatory ventilation (HFOV), as we did not at the time have a protocol in place to deliver SSC if infants were ventilated with HFOV.

### 2.3. Development

**Fig. 1.**
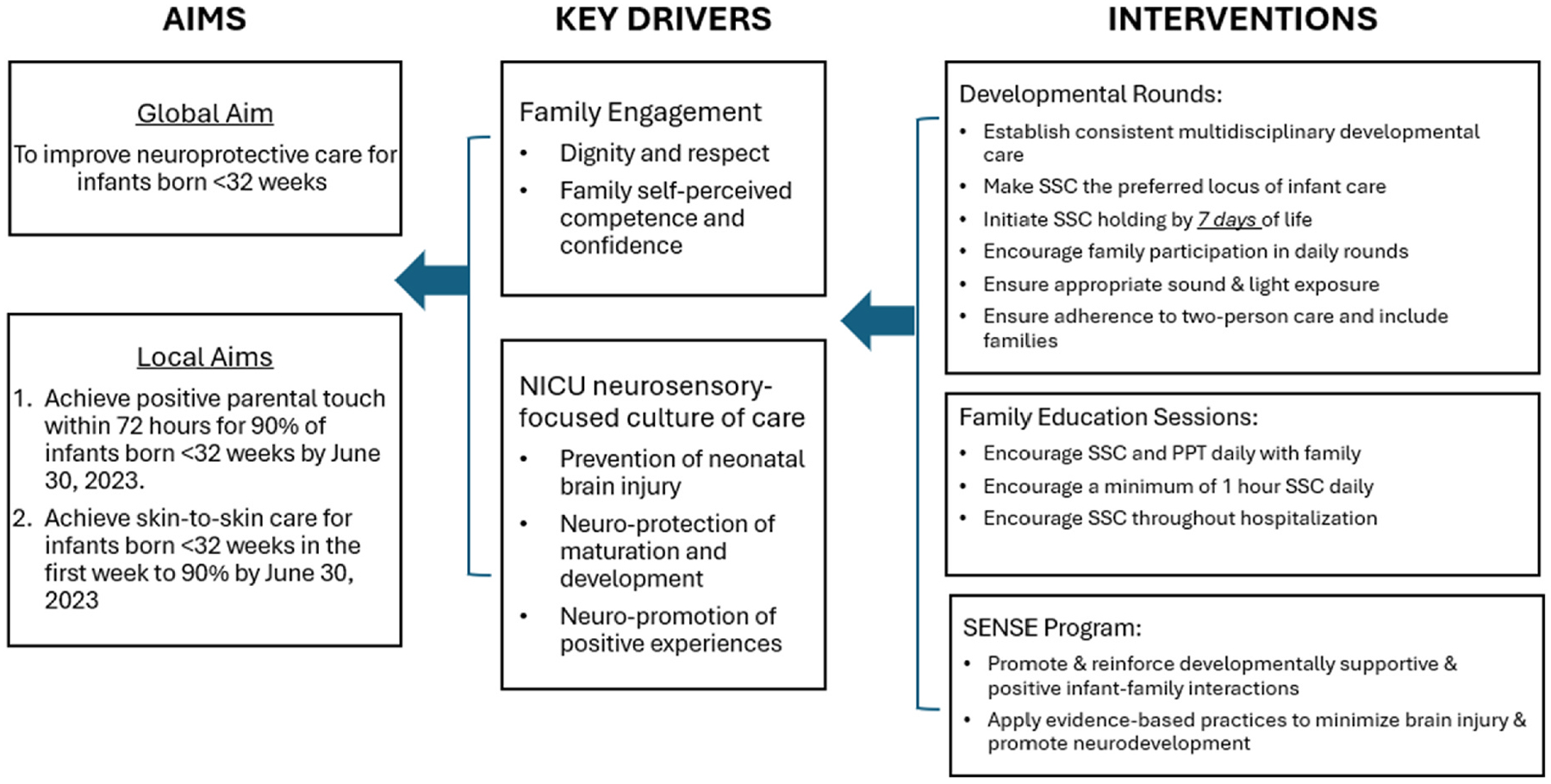
Key driver diagram.

Our QI team met twice monthly to iteratively conceptualize and plan our QI initiative. A key driver diagram (see Fig. 1) was used to organize and plan the initiative (Agency of Healthcare Research and Quality, 2023). We also determined that we needed to evaluate whether the initiative’s effectiveness in achieving early PPT and SSC was sustained one year post-implementation. While PPT is a meaningful activity, it was not included in the clinical workflow or the electronic health record (EHR) at the start of the initiative.

Baseline data were collected from December 2021 to March 2022. Data from April 2022 to May 2022 were not available because only 4 months of baseline data were collected in preparation for the NEOBrain collaborative. There was a two-month gap between the end of the baseline data collection and the start of the initiative.

### 2.4. Implementation

We implemented three interventions through three Plan-Do-Study-Act (PDSA) cycles that focused on increasing education about SSC and PPT among key stakeholders, including medical providers, nurses, other bedside clinicians such as respiratory therapists, and families (Agency for Healthcare Research and Quality, 2024). During the planning stage, we designed our initiative and established that run charts (Institute for Health Improvement, n.d.) would be used to track progress and evaluate success. These run charts tracked the monthly proportion of eligible infants who received SSC within the first week of life and PPT within the first 72 h. In the do stage, we executed the interventions. During the study stage, we reviewed the run chart data and assessed SSC and PPT rates. During the act stage, we used both quantitative data and qualitative stakeholder feedback to refine interventions for subsequent cycles.

### 2.5. PDSA cycle 1: Developmental rounds

We launched weekly multidisciplinary developmental rounds focused on the neurodevelopmental care of each eligible infant as our first initiative intervention in June 2022. These rounds were led by an occupational and physical therapist and aimed to provide education to staff, providers, and families about the importance of developmental care, including family engagement in care and safety protocols for SSC, PPT, and other developmental interventions, such as ensuring appropriate sound and light exposure, adherence to two-person care practices, and active caregiver involvement in the infant’s everyday care. A developmental rounds form was created to organize pertinent information related to this project and to track the completion of rounds as well as patient developmental progress. Since PPT (e.g., hand hugs) was not yet a part of the EHR, we initially used paper documentation during rounds while working towards better systems for capturing this outcome.

### 2.6. PDSA cycle 2: Caregiver education sessions

We implemented caregiver education sessions in November 2022 with the primary purpose of building caregivers’ knowledge and confidence to foster a secure emotional bond between the caregiver and infant. We met with caregivers within the first 72 h of their infant’s admission to the NICU to discuss the benefits of PPT and SSC. During these sessions, caregivers were provided with a family-focused handout, developed by our parent liaison, that outlined the benefits of PPT and SSC and included step-by-step instructions for performing PPT (e.g., hand hugs) and opportunities to practice hand hugs to ensure caregivers felt confident in providing this type of positive touch intervention to their infant. The documentation of PPT (e.g., hand hugs) was added to the EHR at this time.

### 2.7. PDSA cycle 3: SENSE program

We implemented the SENSE program in January 2023 (Pineda et al., 2023). This program provided developmentally appropriate sensory experiences for preterm infants based on their postmenstrual age. The occupational and physical therapists met with caregivers weekly to review individualized sensory recommendations for their infants and place corresponding bedside signs outlining recommended sensory experiences as information for staff and caregivers.

### 2.8. Sustainability

We met monthly until 12 months post-implementation to evaluate whether we were sustaining our SSC and PPT goals and to identify emergent implementation challenges. We continued to track the proportion of infants receiving SSC within the first week of life, PPT within the first 72 h, and the occurrence of developmental rounds monthly using run charts (Lawson et al., 2018). Stakeholder engagement remained central to sustainability efforts, ensuring continued multidisciplinary support for implementation.

### 2.9. Measures and data collection

All measures were collected as part of our QI project from the EHR and a standard documentation form created for developmental rounds. We extracted time to first PPT and SSC from birth as our primary outcome measures. We also extracted patient-specific variables including gestational age, birth weight, race and ethnicity as identified by parent, sex at birth, whether they were inborn (born at UCSF) or outborn (born at an outside hospital), and history of intubation during the first week of life. We also tracked the number of infants who had intraventricular hemorrhage (IVH) within the first week of life and SSC-associated UPE to examine if there were unintended consequences of earlier initiation of SSC and PPT. SSC-associated UPE was defined as occurring during the SSC transfer, during SSC, or within 30 min of transferring the infant back to the isolate.

### 2.10. Analysis

We calculated the proportion of infants a month who received PPT within 72 h and SSC within the first week of life during each phase of our QI initiative. We used Analysis of Variance (ANOVA) and Pearson’s chisquared to compare patient characteristics, PPT within 72 h of birth, SSC within the first week of life, time to first SSC, IVH within the first week of life, and UPE across the development, implementation, and sustainability phases.

This initiative was conducted as a QI project designed to enhance family engagement and promote a neurosensory-focused culture of care in the NICU. In accordance with institutional policies, it was determined that this project did not meet the criteria for human subject’s research and, therefore, did not require review or approval by the institutional review board.

## 3. Results

There were 149 preterm infants born at <32 weeks gestation admitted to the NICU that met the inclusion and exclusion criteria for our QI initiative (see Table 1). Across the development (N = 14), implementation (N = 69), and sustainability (N = 66) phases, infants had similar gestational ages at birth (*p* = .753) and birth weights (*p* = .326); and there also were similar proportions of infants that were inborn (*p* = .086) and with a history of intubation (*p* = .779).

### 3.1. QI initiative development

Baseline data revealed that infants had a mean gestational age of 29.2 weeks and birth weight of 1162.9 g (see Table 1). Most infants were inborn (85.7 %), and 50 % had a history of intubation. Further, 71.4 % of infants born <32 weeks of gestation were receiving skin-to-skin within the first week of life.

### 3.2. Implementation

#### 3.2.1. PDSA cycle 1: Developmental rounds

**Fig. 2.**
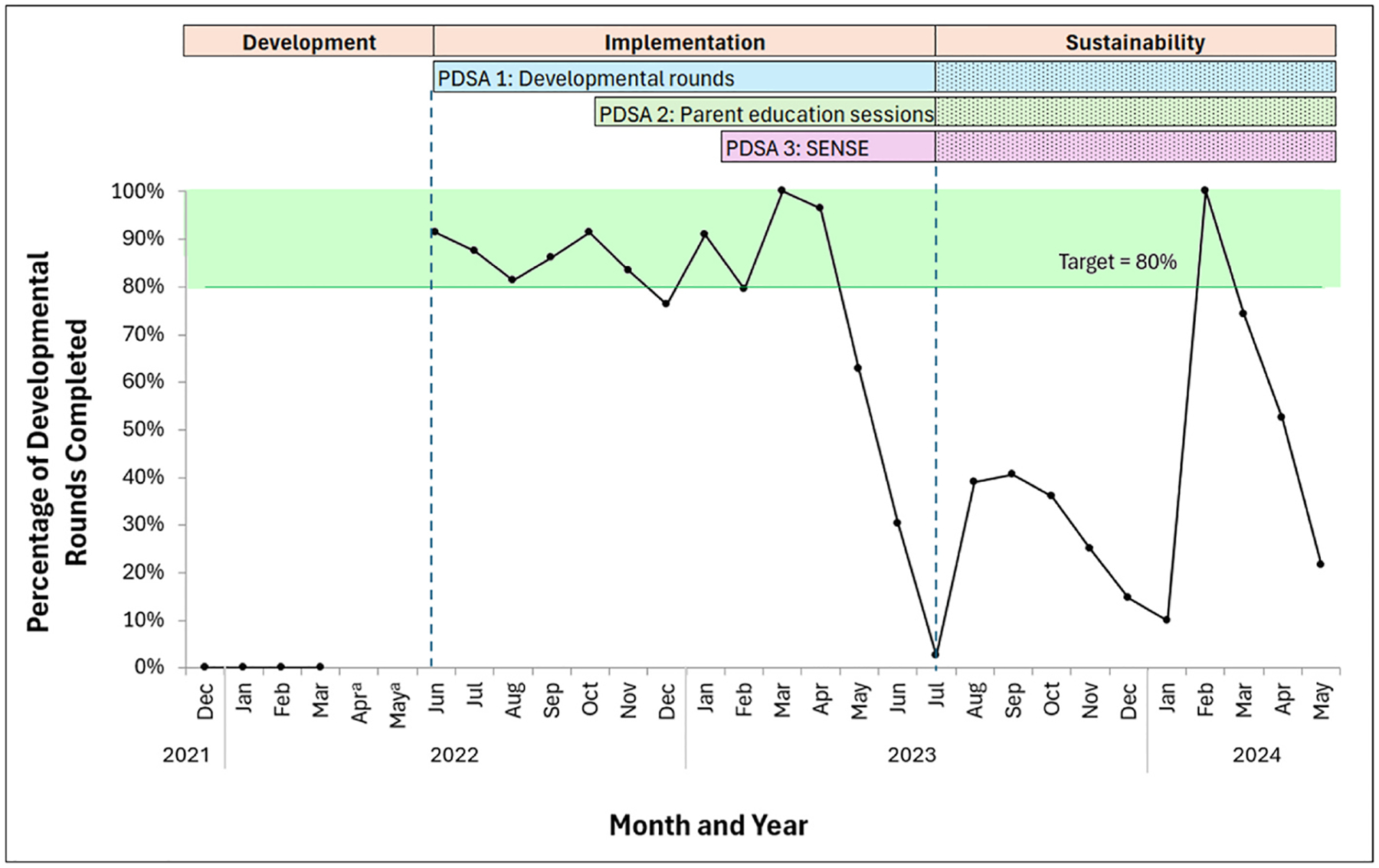
Rate of completion for developmental rounds by month. *Note*. The number of infants per month ranged from 1 to 9. PDSA: Plan-Do-Study-Act. SENSE: Supporting and Enhancing NICU Sensory Experiences. ^a^Data from April and May 2022 are not available, as only four months of baseline data (December 2021 to March 2022) were collected in preparation for the NEOBrain collaborative. A two-month gap occurred between the end of baseline data collection and the start of the initiative.

**Fig. 3.**
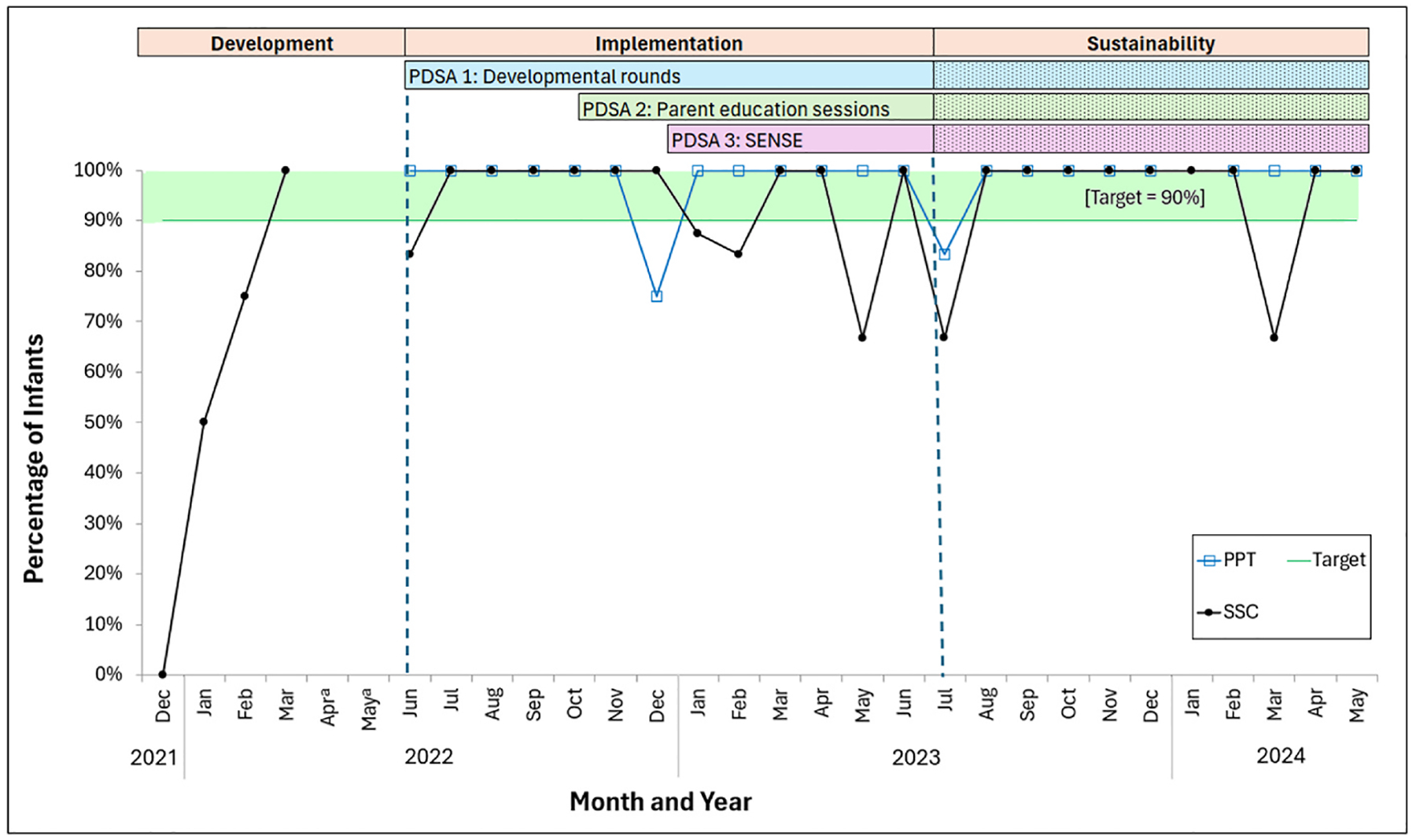
Percentage of Infants a Month with Skin-to-skin Contact in the First Week of Life and Positive Parental Touch in the first 72 Hours of Life by Development, Implementation, and Sustainability Phases of QI Initiative. *Note*. The number of infants per month ranged from 1 to 9. PDSA: Plan-Do-Study-Act. SENSE: Supporting and Enhancing NICU Sensory Experiences. PPT: Positive parental touch. SSC: Skin-to-skin care. ^a^Data from April and May 2022 are not available, as only four months of baseline data (December 2021 to March 2022) were collected in preparation for the NEOBrain collaborative. A two-month gap occurred between the end of baseline data collection and the start of the initiative.

Developmental rounds were completed in 81.3 % of opportunities for eligible infants during the implementation phase (see Fig. 2). SSC rates increased to 100 % and PPT was 100 % in July 2022 after the implementation of developmental rounds (see Fig. 3). One patient had missing PPT data, as it was not recorded either in negative or affirmative in the developmental rounds form, so they were excluded from the PPT analysis.

#### 3.2.2. PDSA cycle 2: Caregiver education sessions

SSC within the first week of life remained at 100 %. PPT dropped from 100 % to 75 % in December 2022. There were no UPE incidents and one IVH diagnosis.

#### 3.2.3. PDSA cycle 3: SENSE program

SSC rates initially decreased with the implementation of this intervention but then increased again to 100 % in March 2023. PPT remained at 100 %. We found three patients who had missing PPT data, as it was unclear who performed the hand hugs and, thus, were excluded from the PPT analysis. There was one UPE incident and no IVH diagnosis.

### 3.3. Sustainability evaluation

**Fig. 4.**
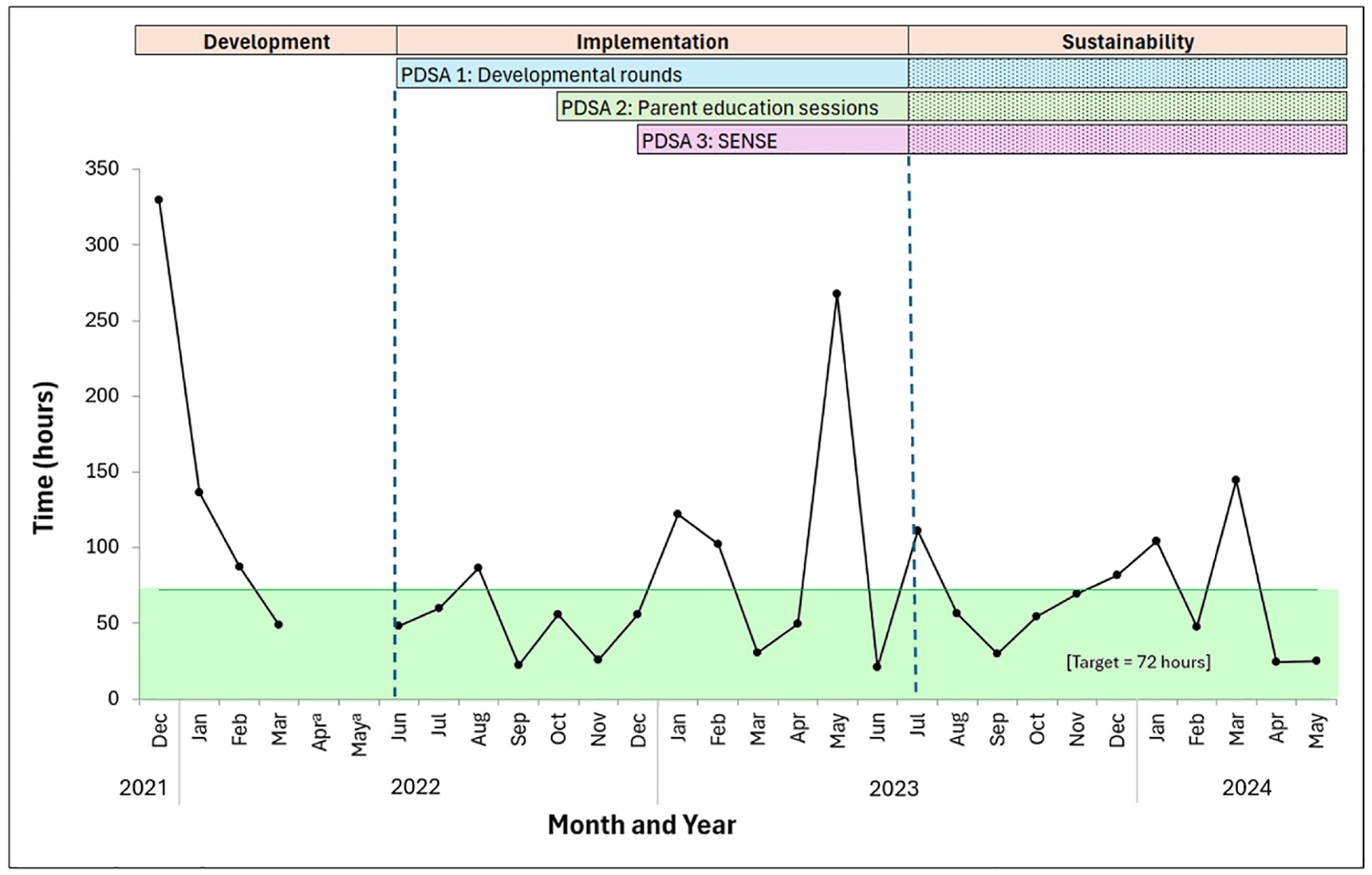
Average time to first skin-to-skin by month. *Note*. The number of infants per month ranged from 1 to 9. PDSA: Plan-Do-Study-Act. SENSE: Supporting and Enhancing NICU Sensory Experiences. ^a^Data from April and May 2022 are not available, as only four months of baseline data (December 2021 to March 2022) were collected in preparation for the NEOBrain collaborative. A two-month gap occurred between the end of baseline data collection and the start of the initiative.

The completion rate of developmental rounds decreased to 37.8 % during the sustainability phase, which coincided with unit staffing changes. There was an improvement in the primary outcome of SSC in the first week of life from 71.4 % during the development phase to 92.8 % with these components of our intervention, which was maintained in the sustainability phase (93.9 %, *p* = .021). Hours to first SSC decreased over the course of the QI initiative, but this was not statistically significant (development: 105.0 h; implementation: 82.7 h; sustainability: 66.1 h; *p* = .285) (see Fig. 4). Most of the infants (92.8 %) received PPT within the first 72 h during the implementation phase, which was maintained during the sustainability phase (93.9 %). Sixteen infants had missing PPT data and were excluded from the PPT analysis.

There were no UPE incidents and one IVH diagnosis during the sustainability phase. Overall, the rate of these potential unintended consequences was not statistically different across the phases of this QI initiative (*p* > .05).

## 4. Discussion

We described our multidisciplinary QI initiative to increase SSC and PPT for very and extremely preterm infants born at <32 weeks gestation. We identified that in the post-COVID peak era in our NICU, only 71 % of our preterm infants born at <32 weeks gestation received SSS and thus developed a QI initiative with three interventions to improve rates of SSC. Upon implementation of our initiative, 92.8 % of preterm infants received SSC within the first week of life, which was sustained after implementation (93.9 %). We also identified an opportunity to implement PPT among these vulnerable infants and found that 98.1 % of our infants received PPT within the first 72 h upon implementation of our initiative, which was sustained after implementation (98 %).

### 4.1. Development

The multidisciplinary composition of our QI team was instrumental to the development and planning of this initiative. By engaging multiple stakeholders early in the process, and in particular our on-staff parent liaison, we identified key barriers to SSC implementation, including staff and caregiver education, buy-in, and motivation, which are consistent with the factors found in the literature (Lee et al., 2012; Suitor, 2023). Thoughtful, multidisciplinary planning facilitated the design of targeted interventions, including developmental rounds, caregiver education sessions, and the SENSE program, each addressing identified challenges through structured education and training. Developmental rounds specifically have been shown to enhance developmental care practices within NICUs, making them a critical component of our initiative (Moss et al., 2023; Muirhead and Bates, 2023)., the literature supports that early caregiver involvement increases caregiver participation and confidence, reinforcing the importance of caregiver education sessions and the SENSE program (Pineda et al., 2021; Whitehill et al., 2021). Thus, careful multidisciplinary planning was foundational in achieving meaningful and lasting improvements in neonatal care.

### 4.2. Implementation

We used the PDSA cycle as the primary framework to iteratively develop, refine, and monitor the implementation of our QI initiatives (Taylor et al., 2014). The iterative nature of PDSA cycles allowed our team to rapidly identify and address emerging challenges, particularly regarding safety concerns associated with SSC. Through continuous data-driven decision-making, we ensured that SSC was safely integrated into routine care without adverse events. This structured, iterative approach facilitated achieving both of our aims of increasing SSC within the first week of life and PPT within 72 h of life for more than 90 % of infants. We also successfully conducted developmental rounds on 81 % of eligible infants, demonstrating intervention uptake and effectiveness.

Although our first PDSA cycle of developmental rounds led to improvements in SSC rates, stakeholder feedback revealed that most caregivers were unavailable during rounds, thereby limiting their access to SSC education. In response, our second PDSA cycle introduced direct caregiver education to increase understanding and participation. As implementation progressed, further feedback highlighted that nurses were not always available to participate in rounds due to clinical demands and that both nurses and caregivers expressed a desire for more structured developmental education. These insights informed our third PDSA cycle, which launched the Supporting and Enhancing NICU Sensory Experiences (SENSE) program as a way to standardize developmental expectations and provide education in a consistent, organized way that could happen at the bedside at any convenient time by the bedside provider (Pineda et al., 2023).

### 4.3. Sustainability

During the 12-month sustainability period, we continued to track SSC, PPT, and developmental rounds using run charts. While SSC rates remained high, completion of developmental rounds declined, likely due to staffing changes, a well-documented barrier to sustaining QI initiatives (Hailemariam et al., 2019). Despite fluctuations in staffing, sustained high SSC rates were likely supported by ongoing education to key stakeholders through caregiver education sessions and the SENSE program, highlighting the importance of ongoing training and early caregiver involvement in sustainment efforts (Burke & Marang-Van De Mheen, 2021; Hailemariam et al., 2019; Pineda et al., 2021; Whitehill et al., 2021). Additionally, the lasting impact of developmental rounds during the initial implementation phase likely facilitated a cultural shift within the unit, reinforcing sustained adherence to SSC practices, a critical factor in maintaining long-term QI improvements (Hailemariam et al., 2019).

To address the observed decline in developmental rounds and strengthen ongoing sustainability efforts, we leveraged continuous collaboration through our partnership with CPQCC. Participating in CPQCC’s structured QI collaborative provided regular opportunities to exchange best practices and strategies for overcoming implementation barriers with other NICUs. Monthly QI team meetings and external support from CPQCC further reinforced the importance of developmental rounds and SSC practices. Recognizing the critical role of developmental rounds in maintaining staff engagement and education, we recently relaunched weekly multidisciplinary developmental rounds in a subset of infants and plan to expand them to all infants under 32 weeks gestation in the coming year. These proactive measures aim to ensure ongoing sustainability and mitigate the impact of future staffing variability (Lawson et al., 2018).

Safety remained a priority given the clinical complexity and medical fragility of our preterm infants. We closely monitored unplanned extubation related to SSC and IVH diagnosis to ensure that there were no unintended effects from our interventions. We did not find any significant differences in pre- and post-QI intervention, reinforcing that early SSC can be safely performed in preterm infants (Bedetti et al., 2023; Kristoffersen et al., 2023; Varty et al., 2023). even among infants on conventional mechanical ventilation and high-frequency jet ventilation. Despite persistent concerns about a potential association between SSC and UPE (Suitor, 2023), SSC has been shown to stabilize and improve respiratory physiology in mechanically ventilated preterm infants (Lee et al., 2022). To expand safe SSC practices, we are currently establishing protocols and safety procedures for SSC in infants receiving HFOV. A future project for us would be to examine the safety and impact of SSC for infants on HFOV on both infant and maternal outcomes as a next step in our initiative to equitably deliver early SSC to all preterm infants.

Overall, our team’s multidisciplinary composition was key to this project’s success, providing diverse perspectives and approaches that contributed to meaningful and impactful interventions. Strong collaboration within our NICU and through CPQCC was instrumental in sustaining intervention momentum, particularly during staffing changes. While we did not evaluate the direct effect of our interventions on length of stay or neurodevelopmental outcomes, these remain important areas for future investigation. Moving forward, our team hopes to focus on other drivers that address the prevention of neonatal brain injury and neuroprotection by decreasing noxious stimuli (e.g., the number of “pokes”).

### 4.4. Limitations

Limitations of this QI study include challenges with data collection, small baseline sample size, and reliance on observational data collected within clinical workflows, which may introduce potential bias. Documentation challenges, especially for PPT, contributed to missing data, as the EHR lacked a designated field to record caregiver involvement. Improving documentation workflows will be essential to ensure long-term adoption and data accuracy.

While our findings offer valuable insights into real-world implementation, generalizability may be limited to NICUs with similar resources. Our success was supported by institutional infrastructure and collaboration with CPQCC. Future efforts should explore strategies for scaling and adapting these interventions in resource-limited NICUs.

## 5. Conclusion

This QI initiative successfully increased SSC rates for preterm infants <32 weeks’ gestation through a structured, multidisciplinary approach. By addressing key barriers through developmental rounds, caregiver education, and the SENSE program, we demonstrated that SSC can be safely integrated into routine care without adverse effects. While sustainability challenges highlight areas for ongoing reinforcement, the initiative fostered a cultural shift toward prioritizing SSC in our NICU. Future efforts will focus on further optimizing SSC practices, improving PPT documentation, and expanding neuroprotective strategies to enhance developmental outcomes for preterm infants.

## Figures and Tables

**Table 1:** Patient characteristics and their outcomes.

	Development phase (n = 14)	Implementation phase (n = 69)	Sustainability phase (n = 66)	*p*-value
**Patient characteristics**				
Gestational age (weeks±SD)	29.2 ± 1.8	28.7 ± 2.5	28.6 ± 2.4	.753
Birth weight (grams±SD)	1162.9 ± 332.4	1234.4 ± 436.2	1131.9 ± 370.4	.326
Inborn, n (%)	12 (85.7 %)	68 (98.6 %)	61 (92.4 %)	.086
H/o intubation, n (%)	7 (50.0 %)	28 (40.6 %)	27 (40.9 %)	.779
Sex at birth, n (%)				.152
Male	9 (64.3 %)	39 (56.5 %)	28 (42.4 %)	
Female	5 (35.7 %)	30(43.5 %)	38 (57.6 %)	
Race and ethnicity^d^, n (%)				
Hispanic	7 (50.0 %)	24 (34.8 %)	19 (28.8 %)	
White	4 (28.6 %)	18 (26.1 %)	16 (24.2 %)	
Asian	3 (21.4 %)	7 (10.1 %)	8 (12.1 %)	
Black	0 (.0 %)	5 (7.2 %)	5 (7.6 %)	
Other^e^	0 (.0 %)	15 (21.7 %)	18 (27.3 %)	
**Outcomes**				
Completed PPT within the first 72 h^f^, n (%)				.088
No		1 (1.5 %)	1 (2.0 %)	
Yes		65 (98.1 %)	49 (98.0 %)	
Completed SSC within the first week of life, n (%)	10 (71.4 %)	64 (92.8 %)	62 (93.9 %)	.021^a^
				.019^b^
				.782^c^
Hours to first SSC (mean ± SD)	105.0 (98.7)	82.7 (140.7)	66.1 (54.6)	.285
IVH diagnosis, n (%)	2 (14.3 %)	2 (2.9 %)	1 (1.5 %)	.053
Unplanned extubation^g^, n (%)	0 (0 %)	1 (3.6 %)	0 (0 %)	.476

*Note*. PPT: Positive parental touch. SSC: Skin-to-skin care. IVH: Intraventricular hemorrhage.

a *p*-value<.05, overall comparison unless otherwise stated.

b Pairwise p-value comparing development and implementation phases.

c Pairwise p-value comparing implementation and sustainability phases.

d *p*-value not calculated due to small cell sizes.

e “Other” includes American Indian or Alaska Native, Native Hawaiian or Other Pacific Islander, multiracial, unknown, and declined to state.

f Data is not available for the baseline group as PPT was not documented until this QI project was implemented. PPT sample size: Implementation (n = 68), Sustainability (n = 50), due to exclusion of cases with missing data.

g Unplanned extubation sample size: Development (n = 7), Implementation (n = 27), Sustainability (n = 27).

## Data Availability

The data used to support the findings of this study is available from the corresponding author upon reasonable request.
